# Integrative analysis of COL6A3 in lupus nephritis: insights from single-cell transcriptomics and proteomics

**DOI:** 10.3389/fimmu.2024.1309447

**Published:** 2024-05-24

**Authors:** Lisha Mou, Fan Zhang, Xingjiao Liu, Ying Lu, Mengli Yue, Yupeng Lai, Zuhui Pu, Xiaoyan Huang, Meiying Wang

**Affiliations:** ^1^ Department of Rheumatology and Immunology, Institute of Translational Medicine, The First Affiliated Hospital of Shenzhen University, Shenzhen Second People’s Hospital, Shenzhen, China; ^2^ MetaLife Lab, Shenzhen Institute of Translational Medicine, Health Science Center, The First Affiliated Hospital of Shenzhen University, Shenzhen Second People’s Hospital, Shenzhen, China; ^3^ Department of Nephrology, Beijing University Shenzhen Hospital, Shenzhen, China; ^4^ Imaging Department, The First Affiliated Hospital of Shenzhen University, Shenzhen Second People’s Hospital, Shenzhen, China

**Keywords:** systemic lupus erythematosus, kidney disease, lupus nephritis, single-cell RNA sequencing, proteomics, COL6A3, biomarkers, diagnosis

## Abstract

**Introduction:**

Lupus nephritis (LN), a severe complication of systemic lupus erythematosus (SLE), presents significant challenges in patient management and treatment outcomes. The identification of novel LN-related biomarkers and therapeutic targets is critical to enhancing treatment outcomes and prognosis for patients.

**Methods:**

In this study, we analyzed single-cell expression data from LN (n=21) and healthy controls (n=3). A total of 143 differentially expressed genes were identified between the LN and control groups. Then, proteomics analysis of LN patients (n=9) and control (SLE patients without LN, n=11) revealed 55 differentially expressed genes among patients with LN and control group. We further utilizes protein-protein interaction network and functional enrichment analyses to elucidate the pivotal role of COL6A3 in key signaling pathways. Its diagnostic value is evaluate through its correlation with disease progression and renal function metrics, as well as Receiver Operating Characteristic Curve (ROC) analysis. Additionally, immunohistochemistry and qPCR experiments were performed to validate the expression of COL6A3 in LN.

**Results:**

By comparison of single-cell and proteomics data, we discovered that COL6A3 is significantly upregulated, highlighting it as a critical biomarker of LN. Our findings emphasize the substantial involvement of COL6A3 in the pathogenesis of LN, particularly noting its expression in mesangial cells. Through comprehensive protein-protein interaction network and functional enrichment analyses, we uncovered the pivotal role of COL6A3 in key signaling pathways including integrin-mediated signaling pathways, collagen-activated signaling pathways, and ECM-receptor interaction, suggesting potential therapeutic targets. The diagnostic utility is confirmed by its correlation with disease progression and renal function metrics of the glomerular filtration rate. ROC analysis further validates the diagnostic value of COL6A3, with the area under the ROC values of 0.879 in the in-house cohort, and 0.802 and 0.915 in tubular and glomerular external cohort samples, respectively. Furthermore, immunohistochemistry and qPCR experiments were consistent with those obtained from the single-cell RNA sequencing and proteomics studies.

**Discussion:**

These results proved that COL6A3 is a promising biomarker and therapeutic target, advancing personalized medicine strategies for LN.

## Introduction

Lupus nephritis (LN) is as a common consequence of systemic lupus erythematosus (SLE), marked by significant mortality rates (1). Approximately 50% of SLE patients are at risk of developing LN in the future (2, 3). Current LN treatment primarily involves immunosuppressive therapy, typically employing cyclophosphamide or mycophenolate mofetil combined with glucocorticoids. However, these treatments come with a host of adverse effects, making treatment adherence challenging and leading to treatment failure and refractory LN (4, 5). Over 10% of individuals with LN develop into end-stage renal disease in ten years (6). Early diagnosis and intervention are vital for reducing end-stage renal disease risk and improving patient outcomes. The identification of novel LN-related biomarkers and therapeutic targets is critical to enhancing treatment outcomes and prognosis for patients.

Renal biopsy, the gold standard for LN diagnosis, can cause invasive damage, necessitating the exploration of non-invasive approaches. Given the systemic nature of SLE, with multiple organs affected and various positive autoantibodies present, gene expression analysis of peripheral blood cells has emerged as a valuable source of LN biomarkers due to the ease of acquisition. Developing reliable LN-related candidate biomarkers from peripheral blood may offer a less invasive and accessible method for diagnosis and monitoring disease progression.

Single-cell RNA sequencing (scRNA-seq) enables us to analyze gene expression profiling at the single-cell level, offering powerful insights into cellular heterogeneity and biological mechanisms (7, 8). ScRNA-seq is also a valuable method in studying autoimmune diseases, including LN, enabling a comprehensive characterization of the cellular landscape involved in immune inflammations (9). This technology can accurately measure gene expression within individual cells, facilitating the analysis of cell heterogeneity between disease and normal states (10).

This study used scRNA-seq and plasma proteomics to discover biomarkers and therapeutic targets in LN. We identified COL6A3 as a key biomarker in LN. By integrating non-invasive protein expression analysis of peripheral blood cells and implementing precision medicine approaches, early and effective intervention strategies can be devised to reduce the burden of LN and its potential progression to end-stage renal disease.

## Methods

### Data processing

ScRNA-seq data of lupus nephritis (LN) patients were obtained from PMID31110316 (11). Seurat was employed for filtering and subsequent clustering (12). The study analyzed renal and skin tissue from 21 patients with LN and 3 healthy controls. The control group consisted of individuals undergoing nephrectomy for kidney transplant donation, from whom biopsy samples of healthy skin and renal tissue were obtained. Cells with RNA feature counts and read count below 100 and 10000 respectly, were excluded as poor-quality cells. The t-distributed stochastic neighbor embedding (t-SNE) algorithm was applied for visualization (13), and batch effect correction was performed using the “RunHarmony” function (14). Cell subtypes were annotated according to cell markers from the original study (11). Differential expression analysis was performed using the Wilcoxon method to identify genes with significant expression differences (DEGs) between groups, setting adjusted *P*-values to 0.05. 

### Patient samples of LN for proteomics analysis

The blood samples of control (systemic lupus erythematosus (SLE) patients without LN) and LN patients were collected from Shenzhen Second People’s Hospital. Enrollment criteria for LN patient inclusion criteria: (1) Persistent proteinuria: >0.5g/day, or > (+++), (2) Cylindruria: May include red blood cells, hemoglobin, granular casts, or mixed casts. Patients were recruited in October 2022. A total of 11 control and 9 LN patients were included. Blood samples were collected with the clinical information including gender, age, SLEDAI, and drug information details of participants are shown in **Table 1**. Participants gave written informed consent and the ethics approval number is 20220824001.

**Table 1:** Baseline demographic and clinical characteristics of LN patients in proteomics analysis.

Clinical information	All (n=20)	SLE w/o LN (11)	LN (9)	t/Z/Fisher	*P* Value
**Demographic**					
Age, years	39 ± 13	34 ± 10	45 ± 13	-2.174	0.043
Female, no. (%)	20 (100.0)	11 (100.0)	9 (100.0)	NA	NA
SLEDAI Score	8 (3,16)	4 (2,8)	15 (8,19)	-2.886	0.004
BMI, kg/m²	20.8 (18.2, 22.4)	19.8 (17.9, 21.6)	24.0 (18.4, 28.5)	-1.899	0.580
Disease duration, months	12.1 (1.0, 118.6)	36.5 (0.7, 121.7)	12.2 (1.0, 139.9)	-0.419	0.675
**Clinical manifestations**					
Musculoskeletal, no. (%)	6 (30.0)	1 (9.1)	5 (55.6)	NA	0.050
Serositis, no. (%)	2 (10.0)	1 (9.1)	1 (11.1)	NA	1.000
Skin rash, no. (%)	6 (30.0)	3 (27.3)	3 (33.3)	NA	1.000
Raynaud’s phenomenon, no. (%)	1 (5.0)	1 (9.1)	0 (0)	NA	1.000
**Laboratory assessment**					
Anti-dsDNA positive, no. (%)	11 (55.0)	5 (45.5)	6 (66.7)	NA	0.406
Anti-Sm positive, no. (%)	8 (47.1)	2 (22.2)	6 (75.0)	NA	0.057
Anti-Ro positive, no. (%)	9 (50)	7 (70.0)	2 (25.0)	NA	0.153
Anti-La positive, no. (%)	3 (15.8)	2 (20.0)	1 (11.1)	NA	1.000
Anti-U1RNP positive, no. (%)	7 (41.2)	3 (33.3)	4 (50.0)	NA	0.637
Anti-β2GPI positive, no. (%)	2 (11.8)	1 (11.1)	1 (12.5)	NA	1.000
aCL positive, no. (%)	2 (11.1)	0(0)	2 (22.2)	NA	0.471
LAC positive, no. (%)	4 (40.0)	3 (50.0)	1 (25.0)	NA	1.000
Anti-CCP, U/mL	11.5 (1.6, 23.6)	11.5 (1.1, 33.4)	9.0 (2.2, 15.0)	-0.356	0.722
RF, IU/L	10.5 (2.1, 25.6)	15.0 (2.9, 41.2)	5.8 (1.4, 20.5)	-0.8	0.424
Leukocyte, 10^9^/L	4.3 (2.7, 6.1)	4.1 (3.5, 6.5)	4.6 (1.9, 5.7)	-0.646	0.518
Lymphocyte, 10^9^/L	1.1 (0.8, 1.6)	1.3 (0.7, 1.7)	1.1 (0.8, 1.3)	-0.532	0.595
Platelets, 10^9^/L	164.5 (123.8, 221.5)	195.0 (151.0, 249.0)	156.0 (110.5, 170.5)	-1.709	0.870
ESR, mm/h	31.5 (13.8, 62.5)	28.0 (13.0, 48.0)	33.0 (14.0, 79.0)	-0.725	0.468
CRP, mg/L	3.0 (1.5, 4.2)	3.8 (1.9, 5.1)	2.2 (1.2, 3.7)	-1.244	0.214
eGFR (mL/min/1.73 m^2^)	126.0 (107.8, 133.8)	131.4 (125.0, 139.8)	106.4 (85.4, 129.5)	-2.317	0.020
Urea, mmol/L	4.5 (3.1, 6.9)	3.6 (2.8, 4.9)	6.9 (4.5, 10.7)	-2.776	0.006
Uric acid, μmol/L	332.5 (261.4, 420.6)	309 (270.1, 371.4)	419.8 (235.7, 459.4)	-0.95	0.342
Cystatin C, mg/L	1.0 (0.8, 1.1)	0.8 (0.7, 1.0)	1.1 (1.0, 1.5)	-2.86	0.004
Retinol-binding protein, mg/L	40.1 (31.9, 45.8)	32.5 (22.2, 40.7)	46.2 (41.4, 56.5)	-2.889	0.004
Hematuria, no. (%)	8 (42.1)	1 (10.0)	7 (77.8)	NA	0.005
Proteinuria, no. (%)	11 (55.0)	2 (18.2)	9 (100.0)	NA	<0.001
Casts, no. (%)	1 (5.3)	0 (0)	1 (11.1)	NA	0.474
Pyuria, no. (%)	3 (15.8)	0 (0)	3 (33.3)	NA	0.087
Urine albumin (g/24h)	0.56 (0.06, 1.75)	0.06 (0.05, 0.1)	1.23 (0.66, 2.45)	-3.334	0.001
Complement C3, g/L	0.6 (0.3, 1.0)	0.8 (0.5, 1.1)	0.3 (0.1, 1.0)	-1.749	0.080
Complement C4, g/L	0.1 (0, 0.2)	0.1 (0.1, 0.2)	0.05 (0, 0.2)	-1.254	0.210
**Medications**					
Prednisolone, no. (%)	19 (95.0)	11 (100.0)	8 (88.9)	NA	0.450
Hydroxychloroquine, no. (%)	16 (80.0)	10 (90.9)	6 (66.7)	NA	0.285
Mycophenolate mofetil, no. (%)	5 (25.0)	2 (18.2)	3 (33.3)	NA	0.617

Descriptive statistics were employed for data presentation: normally distributed quantitative data were expressed as mean ± standard deviation, non-normally distributed quantitative data were presented as median (P25, P75), and binary categorical variables were represented as percentages. w/o, without.

### Preparation of serum samples and LC-MS/MS analysis

Blood samples were obtained from both LN and control (SLE patients without LN) patients in the fasting state. Initially, cellular remnants were eliminated from the serum sample through centrifugation at 12,000 g for 10 minutes at 4°C. Subsequently, the resulting supernatant was carefully transferred to a fresh centrifuge tube. Employing the Pierce™ Top 14 Abundant Protein Depletion Spin Columns Kit, the top 14 high-abundance proteins were efficiently eliminated. Ultimately, the protein concentration was quantified using a BCA kit as per the manufacturer’s stipulations. LC-MS/MS Analysis was performed by PTM BIO company. Differential expression analysis was performed using the Wilcoxon method to identify proteins with significant expression differences (DEPs) between groups, setting *P*-values to 0.05 and fold change value to >1.5.

### Functional enrichment analysis of COL6A3

We performed GO functional annotation and KEGG pathway enrichment analyses on the set of proteins exhibiting differential expression among two groups in proteomics analysis: control (SLE patients without LN), and LN patients.

### Protein-protein interaction network analyses of COL6A3

Potential protein interactions with COL6A3 were collected and integrated using the STRING database (https://string-db.org/). PPI network analysis was conducted with the relevant genes obtained. A confidence score greater than 0.7 was used as the threshold for significance. We then imported relevant data into Cytoscape (v3.8.2) for analysis and visualization. As hub genes, the top 10 nodes, ranked using MCC of cytoHubba, were identified from Cytoscape’s cytoHubba plugins.

### Validation, clinical correlation analysis, and diagnostic value analysis of COL6A3

We performed a validation of COL6A3 expression levels by an independent dataset from the Ju cohort (15). Additionally, we explored the correlation between COL6A3 expression and renal function, particularly focusing on the glomerular filtration rate, among patients with LN. To assess the diagnostic efficacy of COL6A3 in distinguishing LN patients, receiver operating characteristic curve analysis was utilized.

### Immunohistochemistry

Renal samples from control individuals (patients with IgA nephropathy) and LN patients were collected from Beijing University Shenzhen Hospital and is approved by the Research Ethics Committee in Beijing University Shenzhen Hospital (approval number: [2021] No. (038-Extension 2)). Immunohistochemical staining was conducted on paraffin kidney sections using standard procedures. Briefly, kidney sections were blocked with 5% BSA in PBS and then incubated with primary antibodies (anti-Collagen VI alpha 1/2/3, 1:100, R381465, ZEN-Bio, China) for 2 hours at 37°C. After washing the sections with PBS, streptavidin-biotin complex (SA1050, SABC, BOSTER, Wuhan, China) was added and incubated for 30 minutes. Finally, the sections were washed with PBS, and color development was achieved using BCIP/NBT solution, followed by staining with nuclear fast red for 6 minutes. Images were acquired using an optical microscope (NM IL LED, Leica Microsystems, Wetzlar, Germany).

### Real-time PCR analysis

Blood samples were obtained from both LN patients and healthy controls in the fasting state from Shenzhen Second People’s Hospital. Total RNA from the blood was extracted using the SteadyPure Quick RNA Extraction Kit (AG21025, AG, Hunan, China). Total RNA was reverse transcribed into cDNA using a reverse transcription kit (RR036A, Takara, Japan). Real-time PCR was conducted using the SYBR Green mixture kit (QPK-201, TOYOBO, Japan), followed by Real-Time PCR performed on the QuantStudio™ 3 (Thermo Fisher SCIENTIFIC, USA). The primer sequences used are as follows:

GAPDH-F: 5’-AGATCCCTCCAAAATCAAGTGG-3’;
GAPDH-R: 5’-GGCAGAGATGATGACCCTTTT-3’;
COL6A3-F: 5’-CTGTTCCTCTTTGACGGCTCA-3’;
COL6A3-R: 5’-CCTTGACATCATCGCTGTACTGA-3’.

### Statistical analysis

All statistical analyses of single-cell and proteomics data were performed with R (Version 4.3.1). Results of the mRNA and protein expression of COL6A3 were analyzed by Prism (Version 9.4.0). *P* < 0.05 was regarded as statistically significant. The baseline demographic and clinical characteristics of LN patients in the proteomics analysis were analyzed using SPSS (Version 25.0). Descriptive statistics were employed for data presentation: normally distributed quantitative data were expressed as mean ± standard deviation, non-normally distributed quantitative data were presented as median (P25, P75), and binary categorical variables were represented as percentages. Group differences were assessed using various statistical tests, including t-tests, rank-sum tests, and Fisher’s exact probability test. All hypothesis tests were two-tailed, and a *P*-value less than 0.05 was considered statistically significant.

## Results

### Single-cell analysis of lupus nephritis

**Figure 1:**
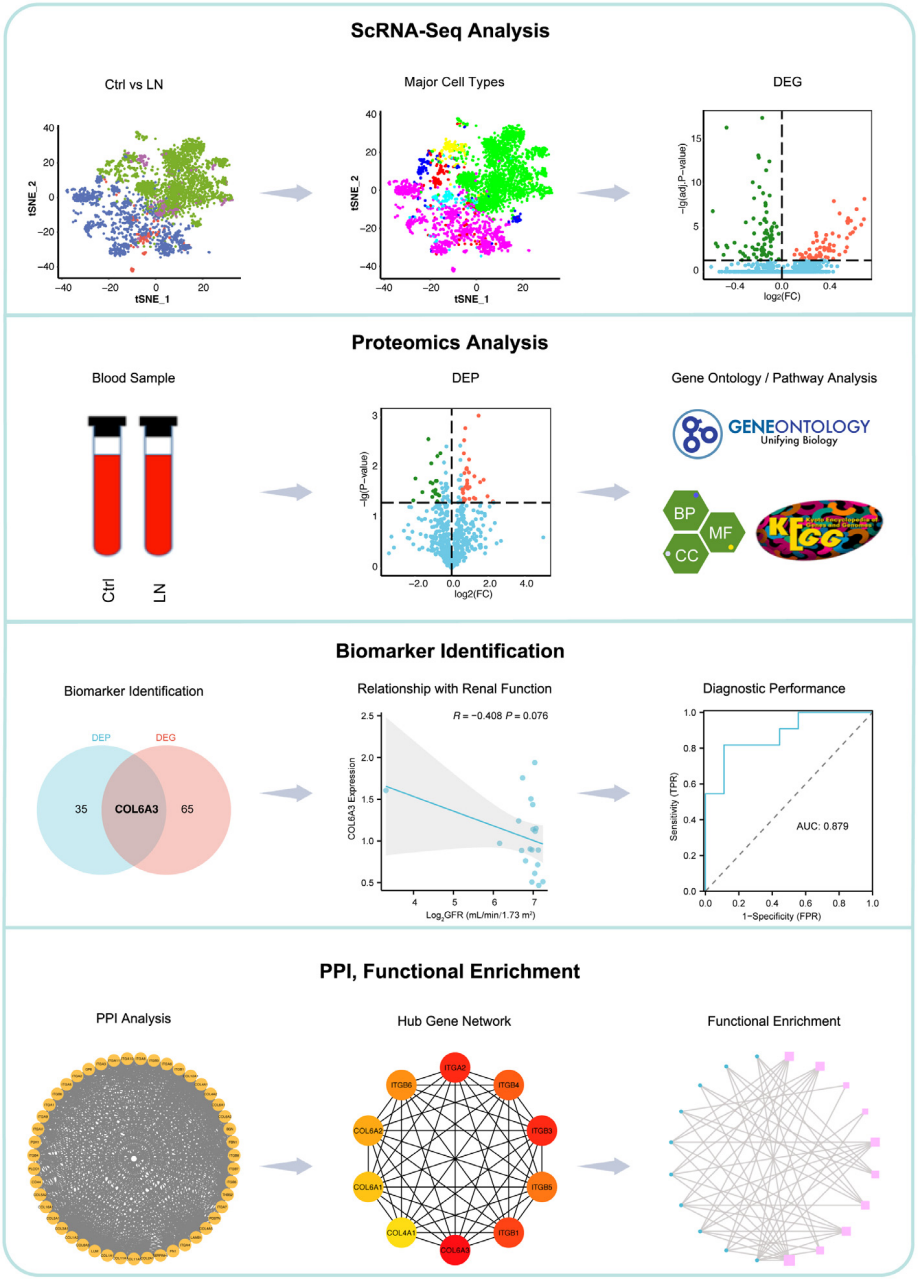
Workflow of this study. We began with single-cell RNA sequencing analysis of 21 lupus nephritis (LN) patients and 3 healthy controls to identify differentially expressed genes (DEGs). This was followed by proteomics analysis to distinguish serum protein expression between SLE patients without LN (n=11) and LN patients (n=9), identifying differentially expressed proteins (DEPs). Gene ontology and pathway analyses of DEPs were conducted. Comparative analysis of DEGs and DEPs leaded to the identification of COL6A3 as a potential biomarker. The association between COL6A3 expression and renal function was evaluated, alongside its diagnostic value using receiver operating characteristic (ROC) curve analysis. Lastly, analyses including protein-protein interaction (PPI), functional enrichment, and gene set enrichment for COL6A3 were performed to elucidate its role in LN.

The workflow of this study is shown in **Figure 1**. We first analyzed the single-cell RNA sequencing data from 21 LN patients and 3 healthy controls, leading to the identification of differentially expressed genes (DEGs). Subsequent proteomics analysis differentiated serum protein expression between control (non-LN SLE patients, n=11) and LN patients (n=9), identifying differentially expressed proteins (DEPs). Further, gene ontology and pathway analyses of DEPs were conducted. Comparative analysis of DEGs and DEPs highlighted COL6A3 as a candidate biomarker. The relationship between COL6A3 expression and renal function was examined, along with its diagnostic potential through receiver operating characteristic (ROC) curve analysis. Lastly, protein-protein interaction (PPI), functional enrichment, and gene set enrichment analyses of COL6A3 were performed, contributing to our understanding of its role in LN.

**Figure 2:**
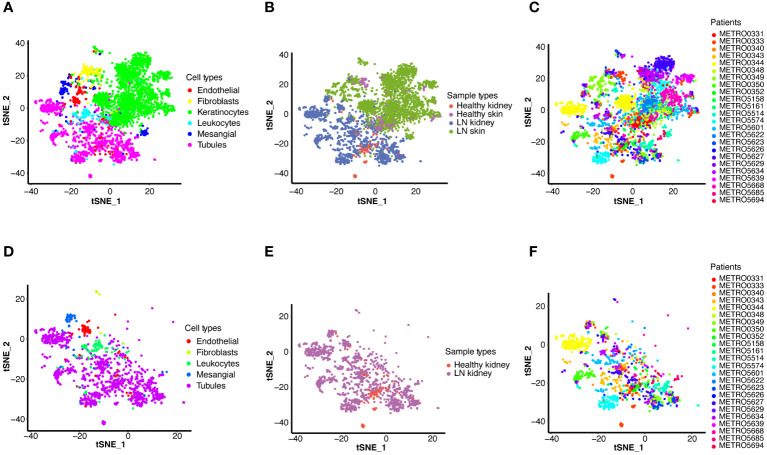
Single-cell RNA sequencing (scRNA-seq) analysis of LN. T-distributed stochastic neighbor embedding (t-SNE) plots display the major cell types in kidney and skin samples from LN patients and healthy controls **(A–C)**, and detailed cell type analysis within kidney **(D–F)**. **(A)** T-SNE analysis of the scRNA-seq data showing six major cell types in the kidney and skin samples. **(B)** T-SNE analysis of two groups, including the kidney and skin samples of healthy control and LN patients. **(C)** T-SNE analysis of the kidney and skin samples from each healthy control and LN patient. **(D)** T-SNE analysis of the scRNA-seq data showing five major cell types in the kidney samples (LN group: n=21, healthy group: n=3). **(E)** T-SNE analysis of two groups, including the kidney samples of healthy control and LN patients. **(F)** T-SNE analysis of the kidney samples from each healthy control and LN patient.

Initially, we accessed a single-cell dataset comprising 21 renal biopsies from LN patients and 3 from control participants without LN, alongside 18 skin biopsies from LN patients and 3 from control participants without LN. Following quality control, normalization, and preliminary dimensionality reduction, t-distributed stochastic neighbor embedding (t-SNE) algorithms were employed to segregate cellular clusters corresponding to both the LN and control cohorts, as illustrated in **Figures 2A–F**. Specifically, **Figures 2A–C** display both renal and skin samples, **Figures 2D–F** focus on renal samples.

In a consistent approach with the initial study, identical cell markers were utilized to identify six principal cell categories (**Figures 2A, D**). In renal samples, tubular cells emerged as the predominant cell type, whereas mesangial cells, leukocytes, endothelial cells, and fibroblasts were identified as minor cell types (**Figure 2D**). Visualization of both kidney and skin sample types via t-SNE is presented in **Figure 2B**. **Figure 2E** showed the kidney samples via t-SNE. Furthermore, t-SNE was also applied to visualize the scRNA-seq results for each patient (**Figures 2C, F**).

**Figure 3:**
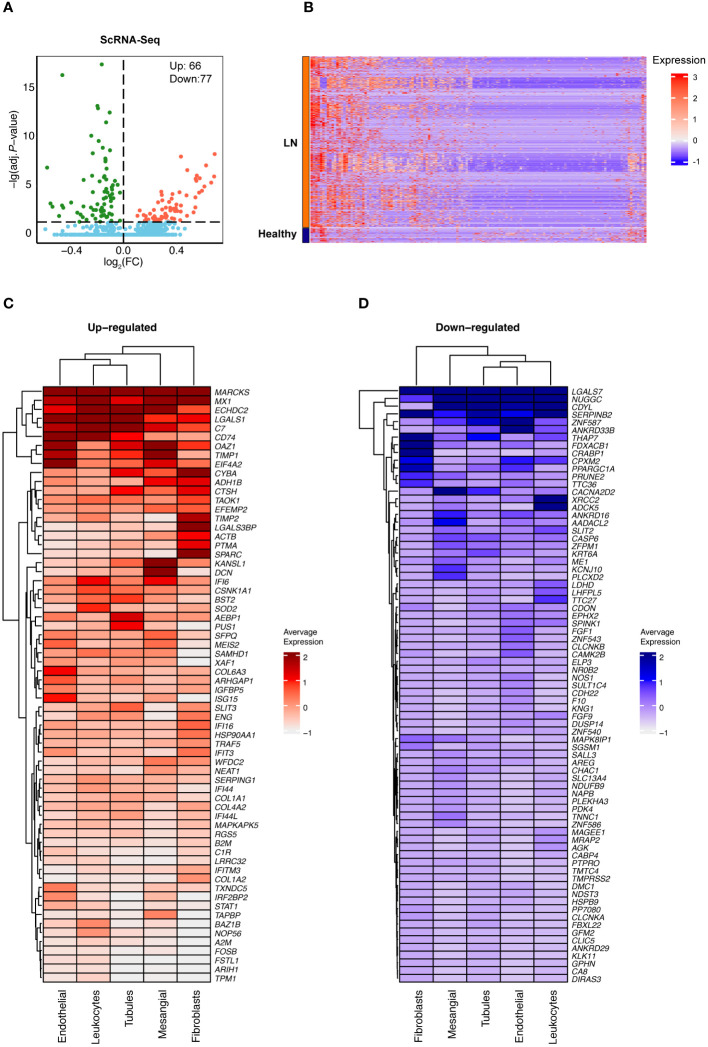
Identification of differentially expressed genes of renal scRNA-seq data in LN. **(A)** A volcano plot showed the upregulated and downregulated DEGs between LN and healthy controls. **(B)** A heatmap showed the upregulated and downregulated DEGs between LN and healthy controls. **(C)** A heatmap showed the upregulated and **(D)** downregulated DEGs between LN and healthy controls in five major cell types.

A total of 143 differentially expressed genes (DEGs) were identified between the LN and control groups, with 66 genes upregulated and 77 downregulated in LN (**Figures 3A–D**). The upregulated and downregulated genes across each cell type were illustrated in the heatmap (**Figures 3C, D**).

### Proteomics analysis, gene ontology and pathway enrichment analyses

**Figure 4:**
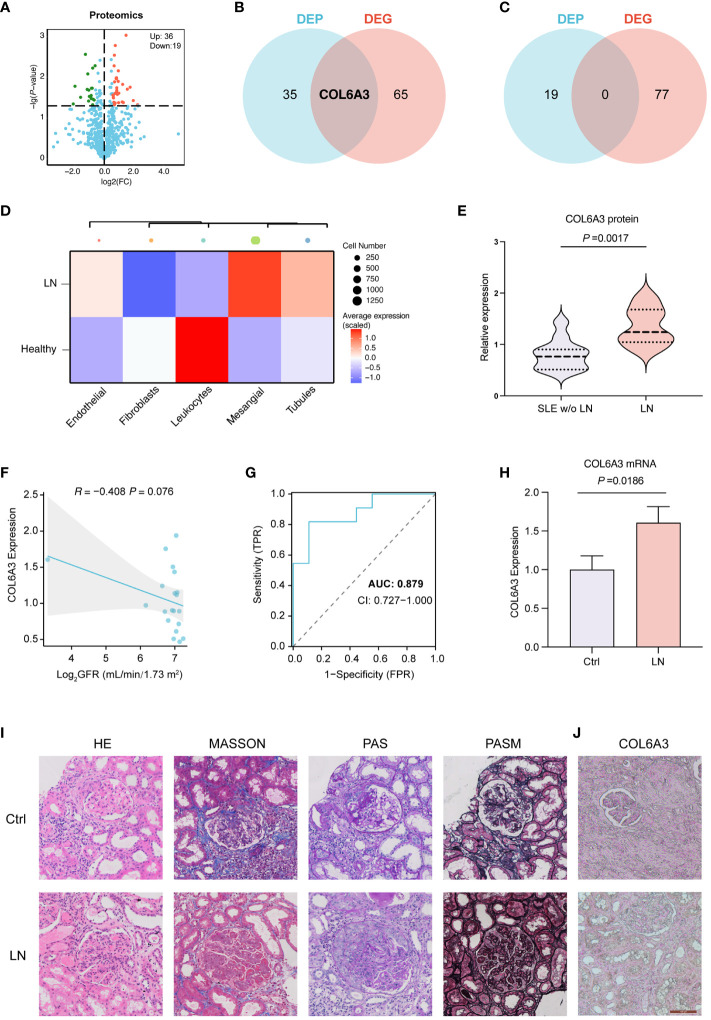
Proteomics analysis of LN. **(A)** Differentially expressed proteins (DEPs) in control (SLE without LN, n=11), and LN patients (n=9). **(B)** Venn diagrams showing the overlap of upregulated DEPs and DEGs, indicating shared and unique molecular features. **(C)** Venn diagrams showing non overlap of upregulated DEPs and DEGs. **(D)** The expression of COL6A3 in five major cell types of renal sample by scRNA-seq analysis. **(E)** The expression of COL6A3 in proteomics data. **(F)** The relationship between COL6A3 expression and estimated glomerular filtration rate (GFR) in our internal cohort. **(G)** The area under the receiver operating characteristic curve (AUC) for COL6A3 in our internal cohort. **(H)** The mRNA expression level of COL6A3 in LN (n=3) and control (healthy volunteers, n=3) examined by Real-time PCR analysis. **(I)** Hematoxylin and eosin (HE), periodic acid-Schiff (PAS), Masson’s trichrome (MASSON), Picrosirius red (PSA), and periodic acid silver methenamine (PASM) staining of LN and control (IgA nephropathy) tissue. **(J)** Protein expression of COL6A3 in LN and control (IgA nephropathy, n=3) tissue analyzed by immunohistochemical staining.

LC-MS/MS was used to identify the proteins in the serum of the patients grouped in control (SLE patients without LN, n=11), and LN patients (n=9). In total, 4,459 peptides were identified by the spectrogram analysis, 923 proteins were identified, and 800 of which were quantifiable. Demographic and clinical characteristics of 11 control and 9 patients diagnosed with LN were shown in **Table 1**. We identified 55 differentially expressed genes (DEPs) among patients with LN and the control group with P < 0.05 and fold change >1.5 (**Figure 4A**; **Supplementary Figures 1A, B**), of which 36 were upregulated and 19 were downregulated (**Figure 4A**; **Supplementary Figures 1A, B**).

GO enrichment analyses revealed that the main biological processes (BP) of DEPs between LN and control group were primarily related to mitotic nuclear division, sister chromatid segregation, and mitotic sister chromatid segregation, whereas the cellular components (CC) term, DEPs were mainly localized in collagen-containing extracellular matrix, platelet alpha granule, and platelet alpha granule lumen (**Supplementary Figure 2**). As for the molecular functions (MF) between LN and the control group, DEPs were primarily related to extracellular matrix structural constituent, microtubule binding, and chemokine activity (**Supplementary Figure 2**). Additionally, pathway enrichment analysis showed that DEPs between LN and the control group were primarily related to viral protein interaction with cytokine and cytokine receptor, ECM-receptor interaction, complement, and coagulation cascades (**Supplementary Figure 2**).

### Comprehensive analysis and clinical significance of COL6A3 in LN

We conducted a comparative analysis between the 55 DEPs identified via proteomics analysis and the 143 DEGs discerned between LN and control samples through scRNA-seq analysis. Notably, COL6A3 was found to be upregulated in both the scRNA-seq and proteomics datasets, while there were no intersections found between the downregulated DEPs and DEGs (**Figures 4B, C**). To elucidate the distribution pattern of the central gene COL6A3, we measured its average expression levels within the scRNA-seq cohort. The analysis revealed that mesangial cells displayed the highest expression levels, underscoring their significance in LN pathology (**Figure 4D**). This evidence underscores the critical role of *COL6A3* in mesangial cells during LN progression by scRNA-seq analysis. The proteomics dataset also demonstrated the expression profile of COL6A3 (**Figure 4E**). Further analysis of the relationship between the protein expression of COL6A3 and renal function in our internal cohort revealed a negative correlation with the estimated glomerular filtration rate (GFR) (**Figure 4F**). Moreover, the area under the receiver operating characteristic curve (AUC) for COL6A3 was calculated, yielding a value of 0.879 within our internal cohort, indicating high diagnostic accuracy of COL6A3 in predicting LN (**Figure 4G**). We conducted qPCR experiments to validate the mRNA expression of COL6A3 (**Figure 4H**). The results were consistent with those obtained from the scRNA-seq and proteomics studies. Additionally, histological staining, including HE, PAS, MASSON, and PSAM, of LN and normal renal tissue revealed significant differences between LN and control patients (IgA nephropathy patients) (**Figure 4I**). Furthermore, immunohistochemistry showed a significant difference in COL6A3 expression in renal tissue between the normal and LN groups (**Figure 4J**).

**Figure 5:**
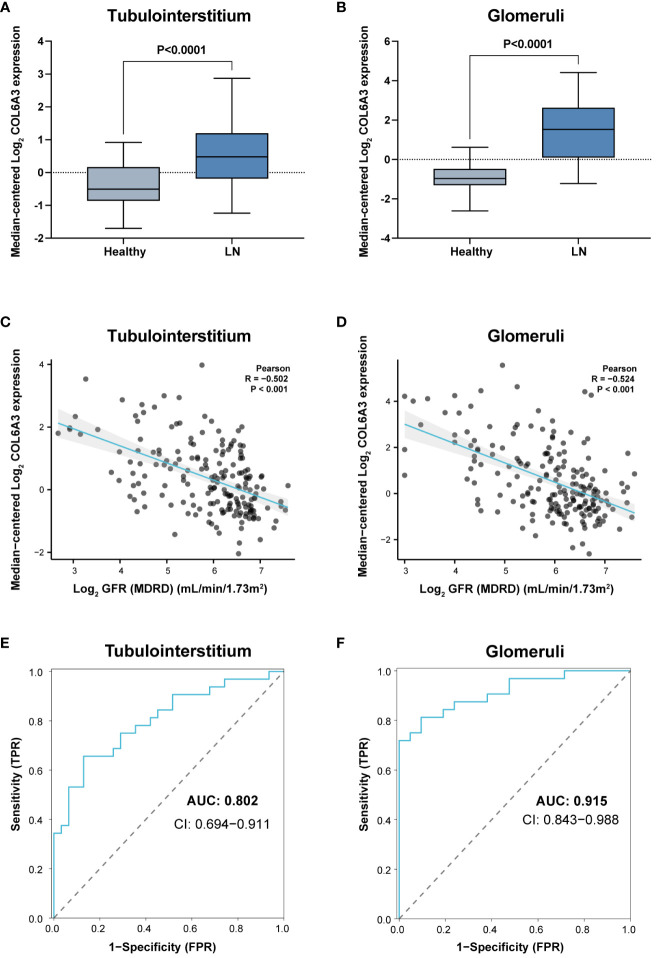
External validation of the clinical significance of COL6A3. Expression in tubulointerstitium **(A)** and glomeruli **(B)** from the Ju cohort (15) and its relationship with GFR **(C, D)**. **(E, F)** Diagnostic performance of AUC results in the Ju cohort.

To further validate the link between *COL6A3* and clinical features, we utilized an external cohort from the Ju cohort (15). Here, the mRNA expression levels of *COL6A3* were significantly elevated in LN patients compared to healthy controls (**Figures 5A, B**), corroborating the negative correlation between *COL6A3* expression and estimated GFR (**Figures 5C, D**). Additionally, the ROC analysis demonstrated the high diagnostic capability of *COL6A3* (AUC=0.802 in tubular samples and AUC=0.915 in glomerular samples) (**Figures 5E, F**), aligning with our internal findings.

### The PPI, functional enrichment, and gene set enrichment of COL6A3

A PPI network was then constructed based on 50 genes closely related to COL6A3 (**Supplementary Figure 3A**). In terms of hub genes, COL6A3 ranked first, followed by ITGA2, ITGB3, ITGB1, ITGB4, ITGB5, ITGB6, COL6A2, COL6A1, and COL4A1 (**Supplementary Figure 3B**). GO and KEGG enrichment analyses were performed on these genes. As a result of these results, these hub genes primarily play a role in integrin-mediated signaling pathway, collagen-activated signaling pathways, and ECM-receptor interaction (**Supplementary Figure 3C**).

## Discussion

In exploring the complexities of lupus nephritis (LN), our study embraced an integrative methodology combining single-cell transcriptomics and plasma proteomics. We identified COL6A3 as a prominent biomarker, with a consistent upregulation observed across both methods. This concordance underscores the potential of COL6A3 in elucidating the molecular landscape of LN. Notably, the marked upregulation of COL6A3 in mesangial cells, as revealed through scRNA-seq, underscores its pivotal involvement in the disease’s pathogenesis and opens potential avenues for targeted therapeutic interventions.

The advent of scRNA-seq technology has revolutionized our capacity to systematically examine cell heterogeneity and identify pathogenic cell populations, thereby enriching our understanding of autoimmune diseases’ underlying mechanisms (9, 16, 17). In the context of LN, recent scRNA-seq endeavors have delineated the intricate immune cell networks (18, 19), underscoring the significance of cytokine expression profiles in mediating renal immune activity (20). This study leveraged high-throughput single-cell data to unearth biomarkers predictive of LN severity and treatment response, setting the stage for personalized therapeutic strategies.

COL6A3, encoding Collagen Type VI Alpha 3, functions primarily as a cell-binding protein, playing a pivotal role in the extracellular matrix’s structural integrity and cellular interactions (21, 22). The observed inverse relationship between COL6A3 expression and estimated glomerular filtration rate (GFR) accentuates its utility in tracking disease progression. Furthermore, the diagnostic potential of COL6A3 affirmed through the area under the receiver operating characteristic curve analysis, advocates for its inclusion in non-invasive diagnostic modalities. External cohort validation, facilitated by the Ju cohort (15), not only reinforces COL6A3’s diagnostic credibility but also exemplifies the merit of cross-cohorts analyses in biomarker substantiation.

Investigation into the protein-protein interaction (PPI) network and the functional enrichment of COL6A3 revealed its engagement in key signaling pathways, implicating integrins, collagen, and extracellular matrix (ECM) receptor interactions. These findings suggest therapeutic intervention targets, offering fresh perspectives for LN treatment strategies.

It’s important to note that while COL6A3 expression was identified predominantly in mesangial cells via scRNA-seq analysis, its presence in the bloodstream, as shown in proteomics data, points towards its systemic impact, including potential roles beyond the mesangium. COL6A3’s subcellular localization in the endoplasmic reticulum and vesicles, indicating a secretory pathway, suggests its ability to be secreted into the circulation, thus impacting LN pathogenesis beyond its site of predominant expression. This secretory nature allows COL6A3 to act both locally within the kidney and systemically, which might explain its detection in blood samples and its involvement in the broader pathophysiological processes of LN.

The expression of COL6A3 and its association with immune cells, particularly M2 macrophages, has been documented in other immune-mediated diseases, indicating its broad role in immune regulation and tissue remodeling (23). Such associations have been noted in conditions like diabetic retinopathy (23) and diabetic nephropathy (24), suggesting the potential of COL6A3 as both a biomarker and therapeutic target across various diseases.

However, the profibrotic nature of COL6A3, as highlighted in its association with deteriorating kidney function in diabetic nephropathy (25), aligns with its observed role in LN, suggesting a shared pathological pathway across kidney diseases. This underscores the rationale for exploring COL6A3 as a therapeutic target in a broader spectrum of kidney conditions. Moreover, the role of COL6A3 in metabolic processes, including inflammation, obesity, and insulin resistance (26, 27), extends its relevance beyond specific organ pathology, suggesting its potential involvement in the metabolic complications experienced by SLE patients.

Our findings, along with related literature, reinforce the significance of COL6A3 as a biomarker and therapeutic target in LN, prompting further research into its roles across various kidney diseases and immune regulation processes. Understanding the function of COL6A3 in the ECM, its influence on immune cell activity, and its contribution to fibrotic processes could inform the development of treatments targeting the intricate interplay of immune and fibrotic mechanisms in LN.

Nevertheless, the limitations of this study, including the reliance on available datasets and the complex pathophysiology of LN, necessitate further investigation into the role of COL6A3 in diverse and larger patient cohorts. Overcoming these challenges and translating biomarker discoveries into clinical practice will require the development of standardized assays and a deeper understanding of the biomarker’s role in the multifactorial landscape of LN.

In conclusion, this study marks a significant contribution to LN research by pinpointing COL6A3 as a promising biomarker and therapeutic target, offering valuable insights into the molecular underpinnings of disease. Future research is imperative to validate these findings and investigate the clinical translational potential of COL6A3, with an aim towards enhancing diagnostics and therapeutic solutions for LN, ultimately improving patient care and outcomes.

## Data Availability

The datasets presented in this study can be found in online repositories. The names of the repository/repositories and accession number(s) can be found below: https://www.iprox.cn//page/project.html?id=IPX0006998000, IPX0006998000. The mass spectrometry proteomics data have been deposited to the ProteomeXchange Consortium (https://proteomecentral.proteomexchange.org) via the iProX partner repository (28, 29) with the dataset identifier PXD052168.

## Supplementary material

Supplementary Figure 1 Differential protein expression between LN and controls. Heatmaps of upregulated **(A)** and downregulated **(B)** DEPs between LN and controls, providing a visual representation of protein expression differences.

Supplementary Figure 2 Gene Ontology (GO) and KEGG pathway enrichment. Analyzes of DEPs to elucidate the biological processes and pathways enriched in LN versus control groups.

Supplementary Figure 3 PPI network and functional enrichment of COL6A3. **(A)** The PPI network was constructed based on 50 genes closely related to COL6A3. **(B)** The top ten hub genes of PPI network. **(C)** Enrichment of GO and KEGG pathways for COL6A3 genes and genes associated with closed interactions.
