# Corrigendum: Integrative analysis of COL6A3 in lupus nephritis: insights from single-cell transcriptomics and proteomics

**DOI:** 10.3389/fimmu.2024.1508292

**Published:** 2024-10-21

**Authors:** Lisha Mou, Fan Zhang, Xingjiao Liu, Ying Lu, Mengli Yue, Yupeng Lai, Zuhui Pu, Xiaoyan Huang, Meiying Wang

**Affiliations:** ^1^ Department of Rheumatology and Immunology, Institute of Translational Medicine, The First Affiliated Hospital of Shenzhen University, Shenzhen Second People’s Hospital, Shenzhen, China; ^2^ MetaLife Lab, Shenzhen Institute of Translational Medicine, Health Science Center, The First Affiliated Hospital of Shenzhen University, Shenzhen Second People’s Hospital, Shenzhen, China; ^3^ Department of Nephrology, Beijing University Shenzhen Hospital, Shenzhen, China; ^4^ Imaging Department, The First Affiliated Hospital of Shenzhen University, Shenzhen Second People’s Hospital, Shenzhen, China

**Keywords:** systemic lupus erythematosus, kidney disease, lupus nephritis, single-cell RNA sequencing, proteomics, COL6A3, biomarkers, diagnosis

In the published article, there was an error in the **Funding** statement. We had missed writing two fundings, Team-based Medical Science Research Program (2024YZZ06), and Shenzhen High-level Hospital Construction Fund (2024). The correct **Funding** statement appears below.

“The author(s) declare financial support was received for the research, authorship, and/or publication of this article. This work was supported in part by the Shenzhen Science and Technology Program (No. JCYJ20190809095811254, No. JCYJ20200109140412476, No. GCZX2015043017281705), Team-based Medical Science Research Program (2024YZZ06), Shenzhen High-level Hospital Construction Fund (2024), the Clinical Research Project in Shenzhen (20213357002 and 20213357028)”.

The authors apologize for this error and state that this does not change the scientific conclusions of the article in any way. The original article has been updated.

