# Supplementary figures and images for: Integrative analysis of COL6A3 in lupus nephritis: insights from single-cell transcriptomics and proteomics
Source: Front Immunol. 2024 May 24;15:1309447. doi: 10.3389/fimmu.2024.1309447 (PMC11157080; doi:10.3389/fimmu.2024.1309447)

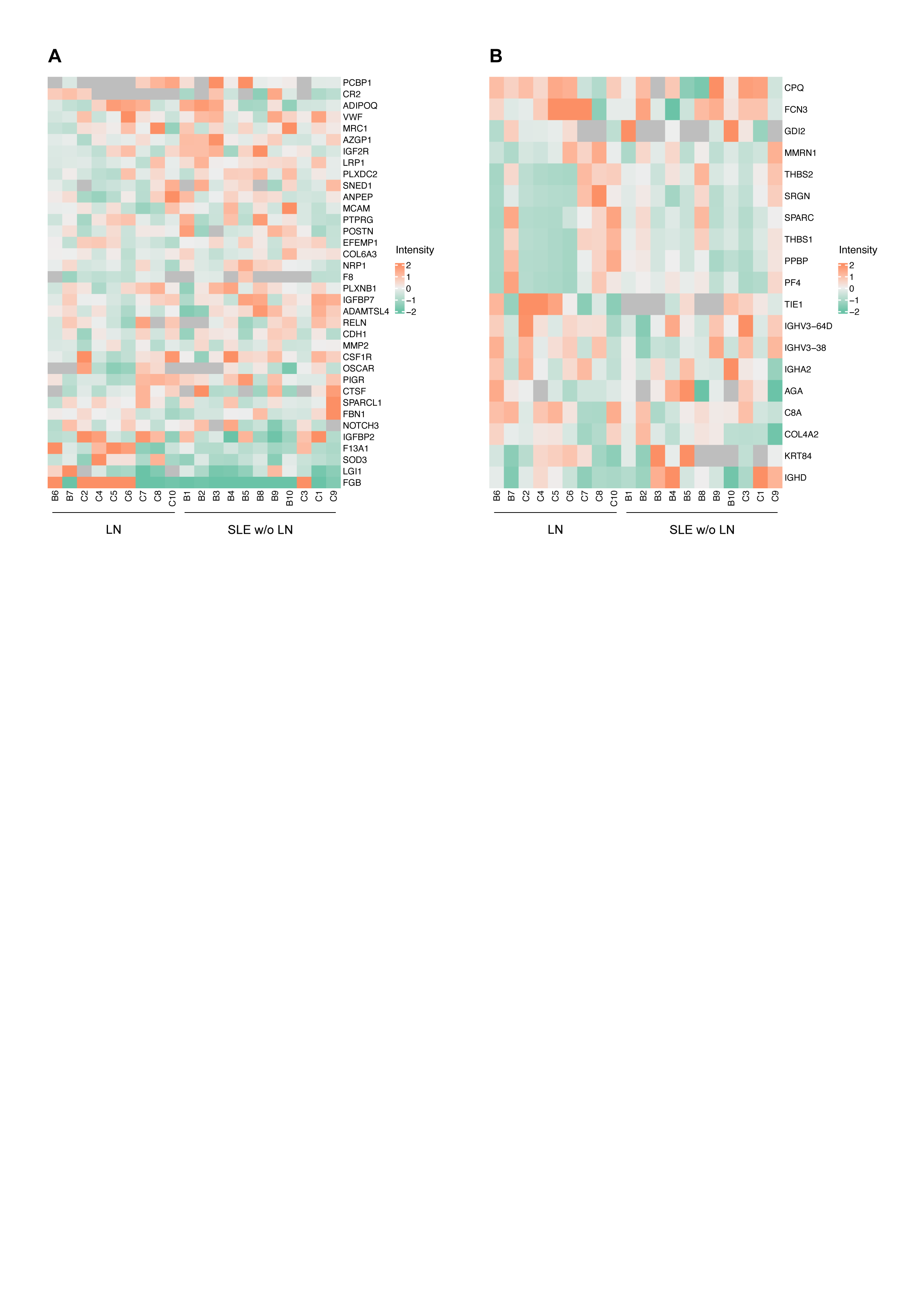

Supplement: Supplementary Figure 1 — Differential protein expression between LN and controls. Heatmaps of upregulated (A) and downregulated (B) DEPs between LN and controls, providing a visual representation of protein expression differences. [file Image1.tif]

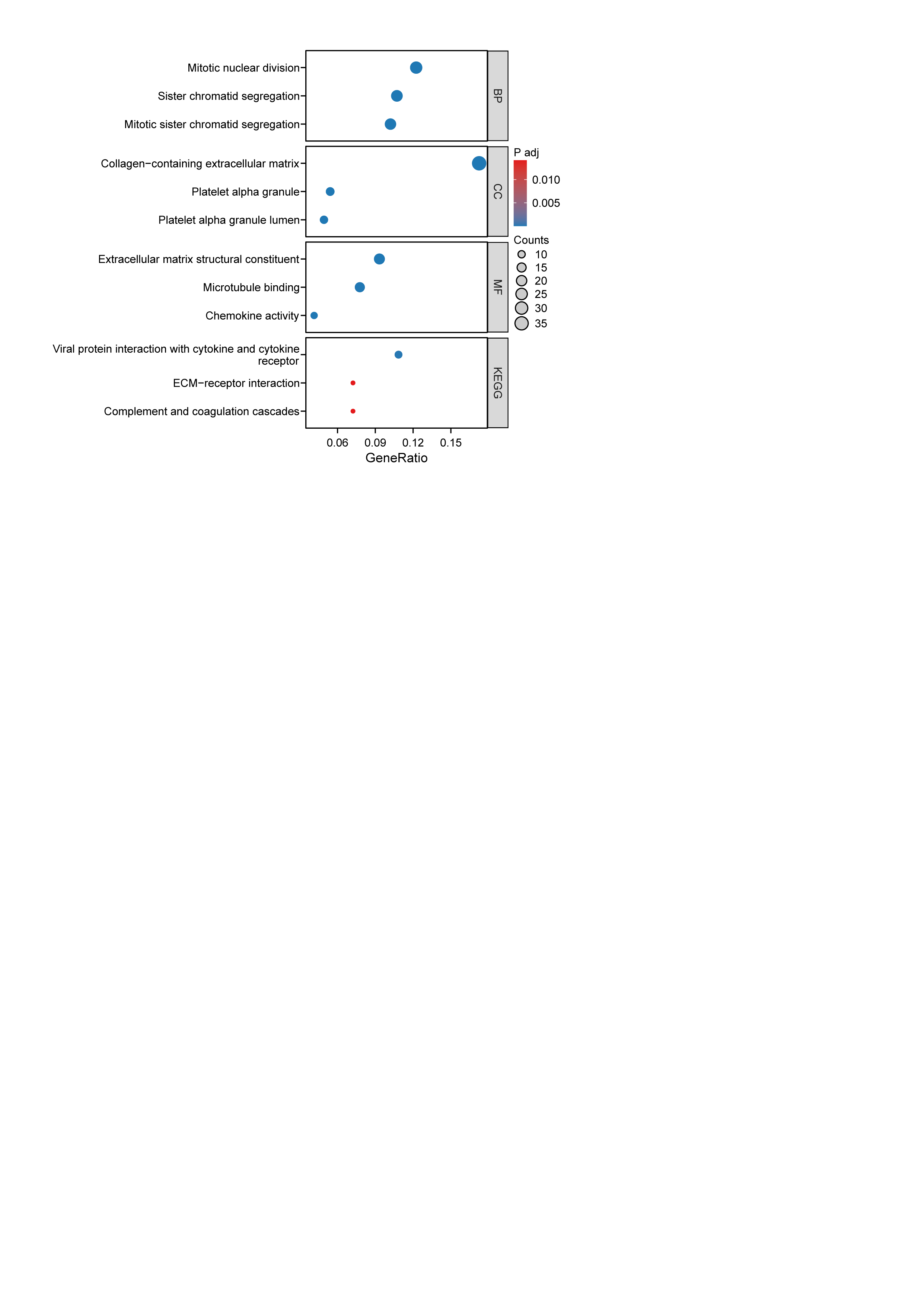

Supplement: Supplementary Figure 2 — Gene Ontology (GO) and KEGG pathway enrichment. Analyzes of DEPs to elucidate the biological processes and pathways enriched in LN versus control groups. [file Image2.tif]

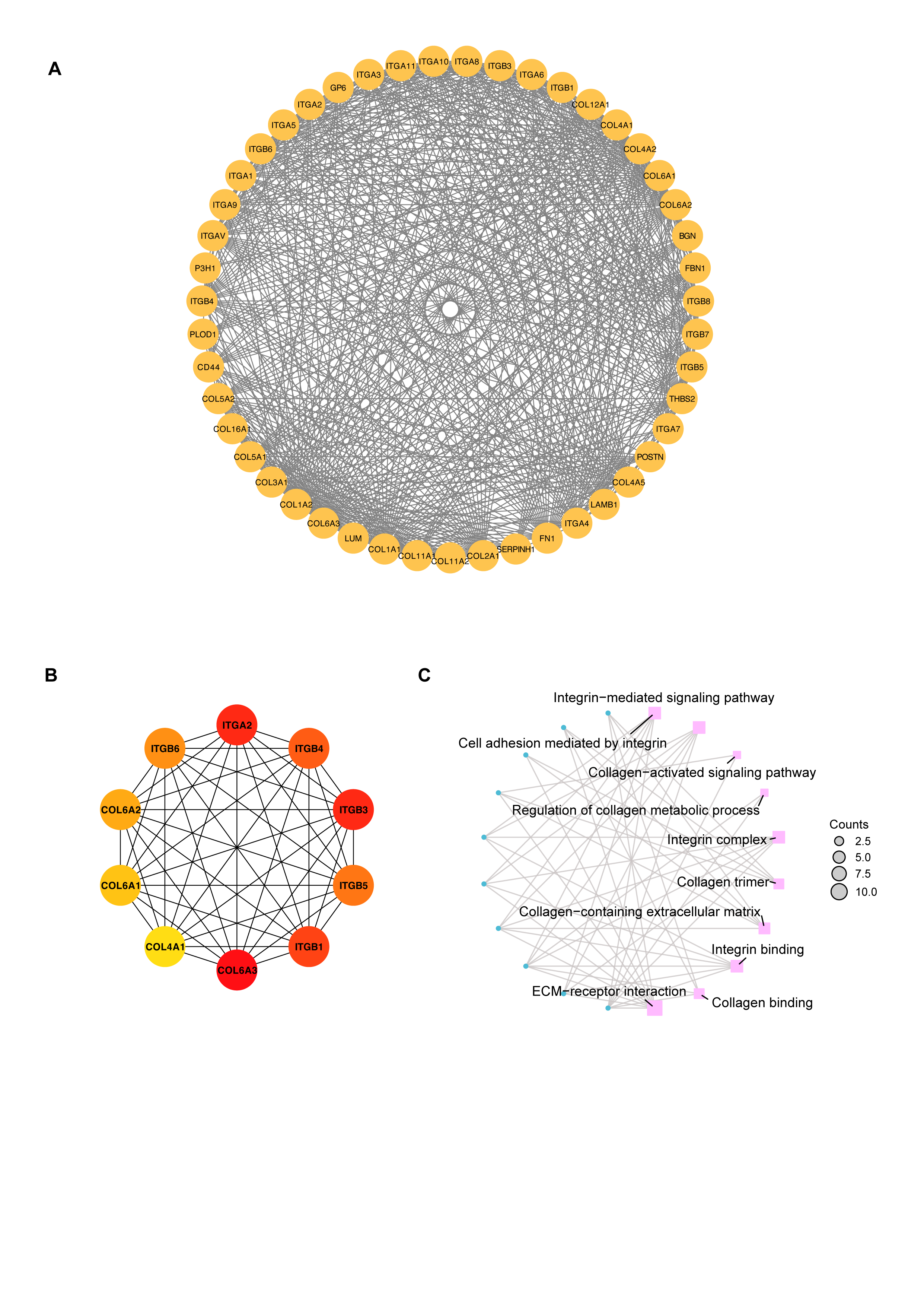

Supplement: Supplementary Figure 3 — PPI network and functional enrichment of COL6A3. (A) The PPI network was constructed based on 50 genes closely related to COL6A3. (B) The top ten hub genes of PPI network. (C) Enrichment of GO and KEGG pathways for COL6A3 genes and genes associated with closed interactions. [file Image3.tif]
